# Gas6/TAM Receptors in Systemic Lupus Erythematosus

**DOI:** 10.1155/2019/7838195

**Published:** 2019-07-09

**Authors:** Philip L. Cohen, Wen-Hai Shao

**Affiliations:** ^1^Section of Rheumatology, Department of Medicine, Temple University, Philadelphia, PA 19140, USA; ^2^Division of Immunology, Allergy and Rheumatology, Department of Internal Medicine, College of Medicine, University of Cincinnati, OH 45267, USA

## Abstract

Systemic lupus erythematosus (SLE) is a multiorgan autoimmune disease associated with impaired immune system regulation. The exact mechanisms of SLE development remain to be elucidated. TAM receptor tyrosine kinases (RTKs) are important for apoptotic cell clearance, immune homeostasis, and resolution of immune responses. TAM deficiency leads to lupus-like autoimmune diseases. Activation of TAM receptors leads to proteolytic cleavage of the receptors, generating soluble forms of TAM. Circulating TAM receptors have an immunoregulatory function and may also serve as biomarkers for disease prognosis. Here, we review the biological function and signaling of TAM RTKs in the development and pathogenesis of lupus and lupus nephritis. Targeting Gas6/TAM pathways may be of therapeutic benefit. A discussion of potential TAM activation and inhibition in the treatment of lupus and lupus nephritis is included.

## 1. Introduction

Systemic lupus erythematosus (SLE) is a chronic autoimmune disease characterized by impairment of the regulation of the immune system and the development of immune-mediated inflammation in multiple organs [1]. Lupus nephritis (LN) is a serious complication requiring aggressive immunosuppression. Despite therapy, about 10% of LN patients develop end-stage renal disease [2]. Defective clearance of apoptotic cells is believed to promote the development of SLE by increasing the availability of potential self immunogens in SLE patients [3]. The TAM (Tyro3, Axl, and Mer) receptor tyrosine kinases (RTKs) are membrane proteins that recognize apoptotic cells with the help of the intermediate molecules, Protein S (ProS) and growth arrest-specific 6 (Gas6) [4–7]. The extracellular part of TAM receptors consists of two Ig-like and two fibronectin-type III domains, which can be proteolytically shed from the cells, forming the soluble forms of TAM receptors [8]. Though serving as classic tyrosine kinase membrane receptors activating proliferation and survival, cell adhesion, and migration in malignant cells, TAM receptors have been implicated in innate and adaptive immunities and have been recently shown to play prominent roles in immune regulation [4].

Gas6 and ProS are vitamin K-dependent TAM ligands that have been studied the most, but other TAM ligands have been reported (Tubby, Tulp1, and Galectin-3) [9–11]. Gas6 and ProS have the same domain structure, with the exception of the thrombin cleavage sites presented in ProS. Gas6 can bind to and activate all three TAM receptors, but ProS only activates Tyro3 and Mer [8, 12, 13]. However, it is worthy of note that Gas6 and ProS are also important regulators of thrombosis and many other biological processes [14]. Gas6 is believed to contribute to platelet aggregation [15]. Deficiency of Gas6 prevents venous and arterial thrombosis [14, 16]. Knockout of ProS and Gas6 leads to the loss of Mer-dependent retinal pigment epithelium phagocytosis in mice [17], suggesting a redundant role of TAM ligands and dominant role of Mer in the phagocytosis of photoreceptors.

Here, we review the current literature on immunobiological function of TAM receptors and their ligands in SLE. We discuss the soluble TAM receptors in the context of disease development and prognosis. Finally, we explore strategies that target TAM receptors in lupus and lupus nephritis. We will focus mainly on the roles of Axl and Mer in lupus and lupus nephritis. Though Tyro3 expression and function primarily associate with the central nervous system [18–20], we will review the published Tyro3 studies under the scope of immune regulation suggesting a function in the pathogenesis/therapeutics in lupus.

## 2. TAM Signaling Pathways and Immunobiological Functions: Implication of Function in SLE

**Figure 1:**
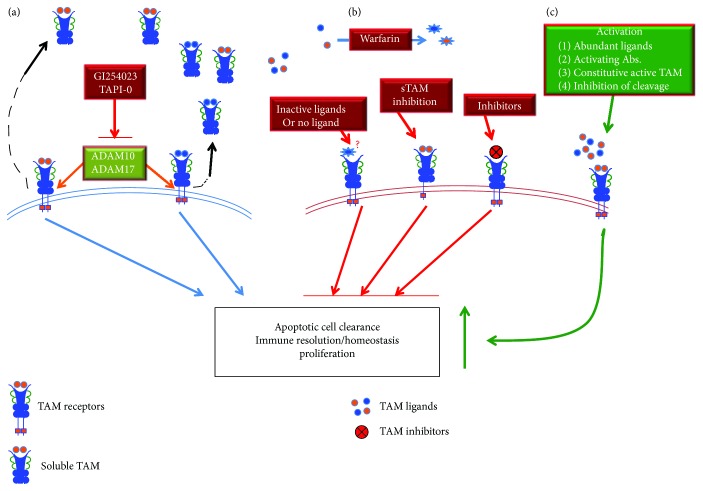
Pathogenic and therapeutic roles of TAM receptors in lupus. (a) Normal TAM functions in lupus are shown in light blue arrows [4–8]. Ligand engagement leads to receptor dimerization and autophosphorylation, which result in the activation of TAM downstream signaling. The effector phase of TAM activation links to apoptotic cell clearance, immune homeostasis, and cell survival/proliferation [8, 21, 35]. TAM activation is reported to be associated with metalloproteinase, ADAM10 and ADAM17, activated cleavage of the receptors [47]. sTAM are released thereby [48–51]. (b) Pathogenic roles of TAM receptors are shown in red arrows. Defects of TAM activation occur in several conditions, including inactivation/exhaustion of the ligands, TAM inhibition, and sTAM-mediated inactivation [48, 54–58]. The consequence of impaired TAM function will be the accumulation of apoptotic debris and breakdown of immune tolerance and autoimmune disease develops over time [36, 39]. (c) Potential TAM-targeted therapeutic roles in lupus are shown in the green box [34, 77–79]. Enhancement of TAM activation can be achieved through exogenous administration with TAM ligands, activating Abs, or inhibition of sTAM generation. Construction of constitutive activated TAM is also on the way.

Activation of the TAM receptors has been shown to affect a diversity of cellular functions, including survival, proliferation, migration, and phagocytosis (Figure 1). Numerous studies of TAM receptor activation and signaling have been published. However, variable outcomes have resulted in an inconsistent understanding of TAM signaling. A thorough investigation of TAM ligand/receptor specificity and optimal activation was undertaken by the Lemke group [21]. Purified Gas6 and ProS are capable of inducing Tyro3 and Mer phosphorylation, which also allow cross-species ligand-receptor activation. However, Axl could be activated only by Gas6 [21, 22]. Most importantly, when different compounds and combinations of ligands and Phosphatidylserine (PtdSer) were compared, maximal activation of the TAM receptors required the simultaneous presence of ligands, PtdSer, and calcium ions [21]. Interestingly, the widely used goat anti-Mer (AF591) and anti-Axl (AF759) antibodies from R&D Systems induced receptor phosphorylation [23], but blocked receptor-mediated phagocytosis of apoptotic cells [24], simultaneously. Nevertheless, Gas6 and ProS are present in the serum at a concentration of 0.2 nM [25] and 350 nM [26], respectively. Axl can be activated by Gas6 at a concentration as low as 1 nM [21]. The microenvironmental concentration of Gas6 may be higher than 1 nM, especially in inflammatory conditions. It is a mystery why TAM receptors are not constitutively activated *in vivo* by their circulating ligands. One mechanism is probably through complex inhibition. Over 60% of ProS is actually bound to C4b-binding protein [26] and all Gas6 is bound to sAxl [25]. On the other hand, optimal TAM activation engages ligand, PtdSer, and calcium, a condition that can be mostly satisfied with the presence of apoptotic cells but can also occur during platelet and endothelial cell activation. The presence of PtdSer on the surface of apoptotic cells is probably the optimal condition for ligand-induced receptor dimerization, which causes a conformational change in the cytoplasmic domain that activates the tyrosine kinase catalytic activity. It may also be possible that low level phosphorylation of TAM receptors by circulating ligands occurs. Such activation may be important for the maintenance of quiescent stage immune homeostasis. However, the exact mechanism demands in-depth investigation.

Much of the early work on TAM signaling pathways was done with chimeric receptors conjugating a TAM receptor intracellular kinase domain to an extracellular receptor domain not normally expressed in the target cells [8]. However, care must be taken when interpreting the data, as multiple factors may contribute to the final outcome of the signaling cascade, including receptor dimerization, extracellular engagement, and ligand/PtdSer complexes in association with the apoptotic cell presence. Most recent work on TAM signaling focuses on the readout of proliferation, migration, and invasion due to a pivotal role of TAM receptors in cancer metastasis, survival, and therapy resistance [27, 28]. Nevertheless, early work by Rothlin et al. demonstrated that TAM receptor signals control the amplification of TLR signaling. The best-known signaling molecules activated by TAM receptors in this scenario are SOCS1/3 [7], as reviewed elsewhere [4, 6, 29]. TAM receptors are potent suppressors of T-cell dendritic cell (DC) responses [30, 31]. However, the signaling cascade has not been worked out. New discoveries have been pointed to distinct and nonoverlapping roles of Axl and Mer in regulating immune responses [32]. Mer is expressed in many cells and functions in the maintenance of immune homeostasis within tissues. Axl expression is inducible and is responsive to inflammatory conditions [32]. Axl activation leads to marked suppression of *Ifn* mRNA in mice injected with anti-Axl antibodies [23], and similar inhibition was also observed in DCs when Axl is activated by Gas6 [7]. Mer was found to be highly expressed on endothelial cells in mouse kidneys [33]. We found that Mer activation leads to the suppression of LPS signaling in primary glomerular endothelial cells through the upregulation of SOCS3 but not SOCS1 [33]. Axl expression in mesangial cells is promoted largely by transcription factor Sp1, but not Sp3. The activation of Axl in mesangial cells links to Akt activation, leading to mTOR phosphorylation [34]. It seems reasonable to conclude that TAM receptors have distinct patterns of expression and disparate signaling and that their function is thus both tissue- and stress-dependent.

TAM receptors play a critical role in regulating innate immunity and maintaining the efficiency of apoptotic cell clearance. TAM receptor-facilitated recognition of apoptotic cells requires the binding of TAM ligands, as bridging molecules, to PtdSer exposed on the surface of apoptotic cells [8, 35]. TAM receptors are of special significance for macrophage and monocyte recognition of apoptotic cells [35–37], a process thought to be impaired in SLE patients [38]. TAM-facilitated phagocytosis of apoptotic cells releases anti-inflammatory cytokines by the phagocytes and induces immune tolerance by supplying autoantigens in a noninflammatory environment [38]. The importance of the involvement of the TAM receptors in the regulation of immunity has been clearly demonstrated in animal models. Mice lacking Mer only (single knockout) suffer from impaired clearance of infused apoptotic cells and go on to develop moderate lupus-like autoimmunity [36]. Mice lacking both Mer and Axl receptors develop more severe lupus-like pathology. Ablation of all three TAM receptors in mice (TAM triple knockout) results in a broad spectrum of autoimmune disease with high titer of autoantibodies and pathologies affecting multiple organs, including the kidney [39].

TAM receptors actively participate in immune regulation. Early studies by Rothlin et al. revealed that TAM receptors mediate an inhibitory role in TLR signaling through a negative feedback mechanism, which occurs via the induction of SOCS1 and SOCS3 [7]. Further research suggests that activated T cells produce ProS, which signals through TAM receptors on DCs to limit the magnitude of DC activation [31]. Among the three TAM receptors, Mer seems to be the most potent as an immune regulation checkpoint. Mer-Fc protein, used to mimic Mer on DCs, suppresses activation of naïve and antigen-specific memory T cells [30]. When the constitutively activated form of Mer-Fc fusion protein was expressed on 293T cells, PD-L1 transcripts and surface expression were increased. PD-L1 is well known for regulating the balance between T cell activation, tolerance, and immunopathology [40]. Mer also plays a critical role in germinal center (GC) apoptotic cell clearance by tangible body macrophages [41]. Prolonged apoptotic cell accumulation in GCs of Mer-deficient mice results in elevated B cell and CD4^+^ T_H_ cell responses, leading to autoantibody production [42]. Tyro3, on the other hand, selectively inhibits type 2 immunity. Accordingly, house dust mite- (HDM-) sensitized Tyro3-KO mice display enhanced type 2 responses, accompanied by increased total and effector memory CD4^+^ T cells and type 2 cytokines (IL-4 and IL-13) [43]. Axl is the least studied TAM receptor in immune regulation. Most of the studies have focused on its role in the survival and proliferative function of cancer cells resistant to therapy [44, 45]. It seems reasonable to assume that Axl is less important in immune regulation, as Axl-KO mice are viable and healthy and have a normal life span with no gross anatomical defects [46]. However, early studies of TAM immunoregulatory functions were achieved in the TAM triple knockout mice or Axl/Mer-double knockout mice [7, 31]. It is possible that the immune regulatory function of Axl is redundant compared to that of Mer and Tyro3. Axl may be important in immune regulation only when Mer is deficient or Mer and Tyro3 are both deficient. It is also possible that Axl and Mer heterodimers are important in regulating immune responses, while Axl homodimers lack this function.

## 3. TAM Ligands and Soluble TAM in SLE Pathogenesis

The heterogeneous features of SLE call for the identification of biomarkers that can quantify disease activity and severity. The extracellular domains (two Ig-like and two fibronectin-III domains) of TAM receptors can be proteolytically cleaved by metalloproteases to yield soluble forms of the receptor (sTAM). A disintegrin and metalloproteinase 10 (ADAM10) and 17 are the two main enzymes responsible for the generation of sTAM [47] (Figure 1). All three TAM receptors are shed from the cells and their soluble forms have been found in plasma, although the exact roles of sTAM remain to be further elucidated. Recent reports have evaluated the plasma concentrations of sTAM and ligands in SLE and SLE nephritis. In general, increased plasma levels of all 3 soluble forms of TAM receptors were reported to correlate with the SLE disease activity index (SLEDAI). However, variable results were reported by different groups.

Among all three TAM receptors, the soluble form of Mer was mostly investigated and constant results were achieved throughout all groups of SLE patients studied. Significantly increased plasma concentration of sMer was reported in SLE patient cohorts from China [48], Sweden [49], UK [50], and Spain [51], compared to age- and sex-matched healthy controls, respectively. These increased plasma sMer levels positively correlated with disease activity and severity measured by the SLEDAI score. Several groups made further association analysis of sMer levels with clinical and serological parameters. A strong association of higher plasma levels of sMer with nephritis was reported by three groups [49, 52, 53]. Zhu et al. studied 108 Chinese SLE patients and found that plasma levels of sMer were significantly elevated in patients with proteinuria compared to those without increased urinary protein [53]. Similarly, Wu et al. found that sMer correlated with the presence of nephritis in a study of 96 Swedish SLE patients [49]. It was subsequently reported that SLE nephritis patients with higher sMer levels tended to suffer from proliferative glomerulonephritis (GN) [52]. Notably, there was a correlation between the concentration of sMer and the presence of autoantibodies [53]. In general, findings pointed to the important function of Mer in macrophage and dendritic cell phagocytosis of apoptotic cells. Increased sMer in the plasma can compete with cell-bound Mer, thus acting as a decoy receptor, resulting in defective phagocytosis, a phenomenon observed in human SLE patients. Excessive apoptotic debris may be a source of self immunogens that together with dangerous stimulating signals released in the process results in autoimmunity.

Significantly elevated concentrations of plasma sAxl in SLE patients were repeatedly reported by different groups to correlate with disease activity and severity in lupus [48, 52, 54, 55] and lupus nephritis [52]. Plasma levels of sAxl followed the same trend as the plasma levels of sMer. Similar functions were also suggested. Soluble forms of Tyro3 have been less studied in SLE patients. Significant positive linear correlations with SLEDAI were reported in two cohorts of SLE patients from Sweden [49] and Spain [51]. However, the increased concentrations of sTyro3 were not related to disease activity parameters (SLEDAI, low C1q, or the presence of nephritis) in Swedish SLE patients [49].

There remain controversies regarding serum levels of Gas6 and ProS in SLE pathogenesis. Recarte-Pelz and colleagues reported a correlation of plasma concentrations of Gas6 and ProS with SLE disease activity, yet Gas6 levels were higher while ProS levels were lower in the SLE patients [51]. Suh et al. found no significant overall differences between the levels of ProS and Gas6 in SLE patients and healthy controls [56]. ProS levels were highly correlated with C3 and C4 levels, and lower ProS levels were found in SLE patients with a history of serositis, neurologic disorder, hematologic disorder, and immunologic disorder [56]. On the other hand, Zhu et al. found that severe SLE patients (SLEDAI ≥ 10) showed significantly lower Gas6 levels [48]. Significantly lower Gas6 levels were associated with shrinking lung syndrome in SLE patients in another study [55]. High Gas6 levels were also observed in SLE patients with GN [52]. Altered but not consistent levels of Gas6 and ProS with disease activity in SLE may reflect the important function of the molecules in regulating thrombosis and inflammation. Gas6 is expressed in many tissues, including capillary endothelial cells, vascular smooth muscle cells, and bone marrow cells [14, 16]. Gas6 acts as an acute-phase reactant and is increased during sepsis and pancreatitis [54]. ProS has a critical function in regulating coagulation. Lower free ProS concentrations in plasma are associated with an increased risk of deep venous thromboembolism [57]. Free ProS acts as a cofactor for activated protein C. Nevertheless, plasma concentrations of Gas6 are approximately 1,000-fold lower than those of ProS [58]. In summary, the significance of plasma levels of Gas6 or ProS in SLE patients is complex and may depend on SLE activity and severity and may also be influenced by other clinical parameters, including lupus disease manifestations (lupus nephritis, vasculitis, arthritis, etc.). We observed significantly lower levels of Gas6 in the serum of Axl-KO nephritic mice compared to the WT nephritic mice. Interestingly, the Axl inhibitor-treated nephritic mice also showed significantly lower serum levels of Gas6 in this study (Shao et al. unpublished data). Taken together, Gas6 may serve as a disease diagnostic biomarker for SLE as increased Gas6 levels correlated with SLE severity. Gas6 may also serve as a biomarker for SLE therapeutics, especially in lupus nephritis.

The exact mechanisms regulating sTAM shedding remain unknown. Nevertheless, the upregulation of sTAM in plasma has been suggested by many studies to serve as a biomarker of disease activity and severity in SLE. It may also serve as a marker for disease prognosis. Hilliard et al. [59] found that Mer expression on monocytes of SLE patients receiving prednisone correlated strongly with the dose of corticosteroid. The potential in vivo functions of the soluble TAM receptors can be speculated as follows: (1) interfere with the TAM-mediated clearance of apoptotic cells and platelet aggregation and (2) form a complex with the ligands to compete with cell-bound receptors, functioning as decoy receptors (Figure 1). These functions have been demonstrated with in vivo experiments. However, it is also possible that sTAM receptors activate cell-bound receptors through the formation of homo- or heterodimers to induce signal transduction pathways. This has not been experimentally approved.

## 4. Function of TAM RTKs in the Kidney

The critical role of TAM receptors in kidney homeostasis was first implied by Graham et al.'s report of strong Mer expression in renal tissues [60, 61]. Excessive circulating levels of sMer, indicating increased systemic shedding, have been recently related to the severity of nephritis in patients with lupus and the rapidity of renal function decline in patients with chronic kidney disease of variable origin [62]. Interestingly, lupus nephritis patients with higher sMer, sAxl, and Gas6 levels tended to suffer from proliferative GN [52]. We were the first to identify the protective role of Mer in a mouse model of lupus nephritis [63]. Mer-KO mice were much more susceptible to antiglomerular basement membrane- (anti-GBM-) induced nephritis than age- and sex-matched WT mice. The early-onset renal damage in Mer-KO mice was associated with increased inflammatory cytokines, excessive apoptotic cells, and massive infiltration with neutrophils [63]. Observations suggest that the primary function of Mer in glomerular endothelial cells is to mediate phagocytosis of apoptotic cells and to attenuate immune responses through modulation of cytokine production.

The Gas6/Axl axis has been recently extensively studied in the kidney. Although Gas6 and Axl are generally not detected in healthy kidneys, they are strongly upregulated on mouse and human glomerular mesangial cells and tubular cells at sites of inflammation [12, 64–66]. Gas6 activation of the mesangial Axl receptor has been implicated in the development of glomerular damage in several GN, including diabetic nephritis, lupus nephritis, and IgA nephropathy [64, 67, 68]. Gas6 is an autocrine growth factor for mesangial cells [69]. Gas6 and its receptor Axl play a critical role in the development of GN. Dysregulation of circulating Gas6 is associated with renal disease and is inversely proportional to renal function [65]. Significantly increased levels of Gas6 and ProS were found in chronic kidney disease patients compared with normal controls [65]. Warfarin and the extracellular domain of Axl inhibit mesangial cell proliferation [67]. However, Gas6 inhibition with warfarin might affect the coagulation cascades and prevent thrombotic events by diminishing coagulation, because the coagulation cascade is activated in severe human and experimental GN [70]. Furthermore, warfarin also inhibits the function of ProS, which is more critical in regulating coagulation and protein C activation. Previous studies using Gas6-KO mice have shown a pathological role for Gas6 in anti-GBM nephritis and streptozotocin-induced diabetic nephropathy [71, 72]. Loss of Gas6 protected against mesangial cell proliferation and glomerular hypertrophy and improved proteinuria and survival [72, 73]. These studies suggest that inhibitors of the Gas6/Axl pathway may be of therapeutic benefit in these forms of renal injury. Our recent publication reported that Axl contributes to anti-GBM antibody nephritis by promoting glomerular mesangial cell survival and proliferation, which leads to glomerular mesangial hypertrophy [74]. We found that Axl activation led to mTOR phosphorylation, which likely contributes to the proliferation of mesangial and tubular cells [34]. The mTOR pathway is a critical contributor to human lupus and lupus nephritis [75]. Targeting mTOR activation through Axl inhibition may provide a safe therapy, since Axl-deficient mice are viable and appear to be normal compared to the WT mice [76]. In contrast, rapamycin suppresses immune function, which may cause serious side effects. The safety of long-term use of rapamycin remains unclear.

## 5. Targeting Axl/Mer in Lupus and Lupus Nephritis

Given the body of evidence implicating TAM regulation, activation, and proteolytic cleavage in lupus and lupus nephritis, it is surprising that the therapeutic focus of TAM receptors has yet to be developed. However, approaches have been implicated by work in several directions (Figure 1). Early findings showed reduced LPS-induced sMer in the bronchoalveolar lavage fluid in mice pretreated with an ADAM17 inhibitor [77]. Mohan's group demonstrated that combined inhibition with ADAM10 and ADAM17 rescues the unresponsiveness of lupus-prone splenocytes to Gas6 [78]. A similar rescued phenotype was observed in human PBMC [78]. Thus, restoration of TAM function by targeting sTAM proteases may be a fruitful therapeutic approach in SLE. Studies conducted in the Rothlin lab showed that the addition of recombinant ProS to the ProS-deficient T cell culture rescued the ability of activated ProS^−/−^ T cells to regulate DC function [31]. Though high concentrations of ProS exist in the plasma, the most is in the form of protein complexes [26], limiting its biological function. Administration of free ProS may lead to an *in vivo* approach to enhance T cell-mediated DC activation suppression. However, large amounts of ProS administration may interfere with endogenous ProS homeostasis, indirectly favoring the environment of protein C activation [15]. On the other hand, the amount of free/active ProS is sufficient to control coagulation and remains relatively constant even in situations of inflammation [57, 79]. Further investigation may be needed when pursing this option. Considering the activating potential of certain polyclonal anti-TAM antibodies from R&D Systems, a better approach would be to engineer the antibody to maximize the activating potential yet diminish the blocking activities. TAM receptors would thus be activated to magnify the anti-inflammatory activities, yet preserve phagocytic function. Nevertheless, TAM functions are rather complex and diverse. TAM-mediated immune suppression and efferocytosis have been adopted by cancer cells to their advantage. Promoting TAM function in lupus for therapeutics could possibly result in an undesired favorable environment for tumor development.

We have demonstrated a critical role for the Gas6/Axl pathway in mouse models of lupus nephritis [34, 74]. Targeting the Gas6/Axl pathway is a promising therapeutic strategy for lupus nephritis [12, 69, 74]. Targeting Axl and Mer in the field of cancer research has shown promise, since Axl and Mer overexpression has been linked to cancer cell metastasis, poor survival, and drug resistance [28, 80]. Studies of Axl and Mer in cancer cells not only advance our understanding of TAM receptor signaling and function but also facilitate application of TAM therapeutics in lupus. Over a dozen Axl-targeted therapeutics have been developed in the last decade [81]. Several of them are in active clinical trials now, including Axl small molecular inhibitors (BGB324, TP0903, AVB-S6-500, etc.) and Axl antibody (CAB-AXL-ADC) (for a complete list and status go to https://clinicaltrials.gov). R428 (also called BGB324) is the most selective small molecule inhibitor of Axl and the first kinase inhibitor designed to specifically target Axl [81]. Pharmacologic studies revealed favorable absorption after oral administration of R428 that was accompanied by a dose-dependent reduction in tumor volume [82–84] and extended survival in a mouse model of metastatic breast cancer [85]. We demonstrated significant efficacy of R428-mediated Axl inhibition, with decreased proteinuria and increased survival in mice with anti-GBM-induced nephritis [34], one of the best models for uncovering the molecular and pathological mechanisms that lead to human lupus nephritis [86].

## 6. Conclusions

TAM receptors are essential for the phagocytosis of apoptotic cells, and TAM activation is associated with immunosuppressive responses. TAM deficiency promotes lupus-like autoimmune diseases in mice. Impaired TAM function is associated with lupus disease activity in humans. Plasma levels of soluble TAM receptors generated by proteolytic cleavage and TAM ligands may serve as potential biomarkers for lupus development and prognosis. Finally, encouraging results have been achieved supporting the therapeutic role of TAM receptors in lupus and lupus nephritis.

## References

[1] Pedersen H. L., Horvei K. D., Thiyagarajan D., Seredkina N., Rekvig O. P. (2015). Murine and human lupus nephritis: pathogenic mechanisms and theoretical strategies for therapy. *Seminars in Nephrology*.

[2] Almaani S., Meara A., Rovin B. H. (2017). Update on lupus nephritis. *Clinical Journal of the American Society of Nephrology*.

[3] Munoz L. E., Lauber K., Schiller M., Manfredi A. A., Herrmann M. (2010). The role of defective clearance of apoptotic cells in systemic autoimmunity. *Nature Reviews Rheumatology*.

[4] Rothlin C. V., Lemke G. (2010). TAM receptor signaling and autoimmune disease. *Current Opinion in Immunology*.

[5] Lemke G., Rothlin C. V. (2008). Immunobiology of the TAM receptors. *Nature Reviews. Immunology*.

[6] Rothlin C. V., Carrera-Silva E. A., Bosurgi L., Ghosh S. (2015). TAM receptor signaling in immune homeostasis. *Annual Review of Immunology*.

[7] Rothlin C. V., Ghosh S., Zuniga E. I., Oldstone M. B. A., Lemke G. (2007). TAM receptors are pleiotropic inhibitors of the innate immune response. *Cell*.

[8] Linger R. M. A., Keating A. K., Earp H. S., Graham D. K. (2008). TAM receptor tyrosine kinases: biologic functions, signaling, and potential therapeutic targeting in human cancer. *Advances in Cancer Research*.

[9] Caberoy N. B., Zhou Y., Li W. (2010). Tubby and tubby-like protein 1 are new MerTK ligands for phagocytosis. *The EMBO Journal*.

[10] Caberoy N. B., Alvarado G., Bigcas J. L., Li W. (2012). Galectin-3 is a new MerTK-specific eat-me signal. *Journal of Cellular Physiology*.

[11] Caberoy N. B., Alvarado G., Li W. (2012). Tubby regulates microglial phagocytosis through MerTK. *Journal of Neuroimmunology*.

[12] Yanagita M. (2004). Gas6, warfarin, and kidney diseases. *Clinical and Experimental Nephrology*.

[13] Graham D. K., DeRyckere D., Davies K. D., Earp H. S. (2014). The TAM family: phosphatidylserine sensing receptor tyrosine kinases gone awry in cancer. *Nature Reviews. Cancer*.

[14] van der Meer J. H. M., van der Poll T., van 't Veer C. (2014). TAM receptors, Gas6, and protein S: roles in inflammation and hemostasis. *Blood*.

[15] Foley J. H., Conway E. M. (2013). Gas6 gains entry into the coagulation cascade. *Blood*.

[16] Angelillo-Scherrer A., de Frutos P. G., Aparicio C. (2001). Deficiency or inhibition of Gas6 causes platelet dysfunction and protects mice against thrombosis. *Nature Medicine*.

[17] Burstyn-Cohen T., Lew E. D., Traves P. G., Burrola P. G., Hash J. C., Lemke G. (2012). Genetic dissection of TAM receptor-ligand interaction in retinal pigment epithelial cell phagocytosis. *Neuron*.

[18] Akkermann R., Aprico A., Perera A. A. (2017). The TAM receptor Tyro3 regulates myelination in the central nervous system. *Glia*.

[19] Blades F., Aprico A., Akkermann R., Ellis S., Binder M. D., Kilpatrick T. J. (2018). The TAM receptor TYRO3 is a critical regulator of myelin thickness in the central nervous system. *Glia*.

[20] Prieto A. L., Weber J. L., Tracy S., Heeb M. J., Lai C. (1999). Gas6, a ligand for the receptor protein-tyrosine kinase Tyro-3, is widely expressed in the central nervous system. *Brain Research*.

[21] Lew E. D., Oh J., Burrola P. G. (2014). Differential TAM receptor-ligand-phospholipid interactions delimit differential TAM bioactivities. *eLife*.

[22] Tsou W.-I., Nguyen K.-Q. N., Calarese D. A. (2014). Receptor tyrosine kinases, TYRO3, AXL, and MER, demonstrate distinct patterns and complex regulation of ligand-induced activation. *The Journal of Biological Chemistry*.

[23] Zagorska A., Traves P. G., Lew E. D., Dransfield I., Lemke G. (2014). Diversification of TAM receptor tyrosine kinase function. *Nature Immunology*.

[24] Shao W. H., Eisenberg R. A., Cohen P. L. (2008). The Mer receptor tyrosine kinase is required for the loss of B cell tolerance in the chronic graft-versus-host disease model of systemic lupus erythematosus. *Journal of Immunology*.

[25] Ekman C., Stenhoff J., Dahlback B. (2010). Gas6 is complexed to the soluble tyrosine kinase receptor Axl in human blood. *Journal of Thrombosis and Haemostasis*.

[26] Dahlback B. (2017). The tale of protein S and C4b-binding protein, a story of affection. *Thrombosis and Haemostasis*.

[27] Wium M., Paccez J., Zerbini L. (2018). The dual role of TAM receptors in autoimmune diseases and cancer: an overview. *Cell*.

[28] Verma A., Warner S. L., Vankayalapati H., Bearss D. J., Sharma S. (2011). Targeting Axl and Mer kinases in cancer. *Molecular Cancer Therapeutics*.

[29] O'Neill L. A. J. (2007). TAMpering with toll-like receptor signaling. *Cell*.

[30] Cabezo´n R., Carrera-Silva E. A., Flo´rez-Grau G. (2015). MERTK as negative regulator of human T cell activation. *Journal of Leukocyte Biology*.

[31] Carrera Silva E. A., Chan P. Y., Joannas L. (2013). T cell-derived protein S engages TAM receptor signaling in dendritic cells to control the magnitude of the immune response. *Immunity*.

[32] Dransfield I., Farnworth S. (2016). Axl and Mer receptor tyrosine kinases: distinct and nonoverlapping roles in inflammation and cancer?. *Advances in Experimental Medicine and Biology*.

[33] Zhen Y., Finkelman F. D., Shao W. H. (2018). Mechanism of Mer receptor tyrosine kinase inhibition of glomerular endothelial cell inflammation. *Journal of Leukocyte Biology*.

[34] Zhen Y., Lee I. J., Finkelman F. D., Shao W. H. (2018). Targeted inhibition of Axl receptor tyrosine kinase ameliorates anti-GBM-induced lupus-like nephritis. *Journal of Autoimmunity*.

[35] Lemke G., Burstyn-Cohen T. (2010). TAM receptors and the clearance of apoptotic cells. *Annals of the New York Academy of Sciences*.

[36] Cohen P. L., Caricchio R., Abraham V. (2002). Delayed apoptotic cell clearance and lupus-like autoimmunity in mice lacking the c-mer membrane tyrosine kinase. *The Journal of Experimental Medicine*.

[37] Lemke G. (2017). Phosphatidylserine is the signal for TAM receptors and their ligands. *Trends in Biochemical Sciences*.

[38] Shao W. H., Cohen P. L. (2010). Disturbances of apoptotic cell clearance in systemic lupus erythematosus. *Arthritis Research & Therapy*.

[39] Lu Q., Gore M., Zhang Q. (1999). Tyro-3 family receptors are essential regulators of mammalian spermatogenesis. *Nature*.

[40] Francisco L. M., Salinas V. H., Brown K. E. (2009). PD-L1 regulates the development, maintenance, and function of induced regulatory T cells. *The Journal of Experimental Medicine*.

[41] Rahman Z. S. M., Shao W.-H., Khan T. N., Zhen Y., Cohen P. L. (2010). Impaired apoptotic cell clearance in the germinal center by Mer-deficient tingible body macrophages leads to enhanced antibody-forming cell and germinal center responses. *Journal of Immunology*.

[42] Khan T. N., Wong E. B., Soni C., Rahman Z. S. M. (2013). Prolonged apoptotic cell accumulation in germinal centers of Mer-deficient mice causes elevated B cell and CD4^+^ Th cell responses leading to autoantibody production. *Journal of Immunology*.

[43] Chan P. Y., Silva E. A. C., de Kouchkovsky D. (2016). The TAM family receptor tyrosine kinase TYRO3 is a negative regulator of type 2 immunity. *Science*.

[44] Rankin E., Giaccia A. (2016). The receptor tyrosine kinase AXL in cancer progression. *Cancers*.

[45] Shen Y., Chen X., He J., Liao D., Zu X. (2018). Axl inhibitors as novel cancer therapeutic agents. *Life Sciences*.

[46] Axelrod H., Pienta K. J. (2014). Axl as a mediator of cellular growth and survival. *Oncotarget*.

[47] Weinger J. G., Omari K. M., Marsden K., Raine C. S., Shafit-Zagardo B. (2009). Up-regulation of soluble Axl and Mer receptor tyrosine kinases negatively correlates with Gas6 in established multiple sclerosis lesions. *The American Journal of Pathology*.

[48] Zhu H., Sun X., Zhu L. (2014). Different expression patterns and clinical significance of mAxl and sAxl in systemic lupus erythematosus. *Lupus*.

[49] Wu J., Ekman C., Jonsen A. (2011). Increased plasma levels of the soluble Mer tyrosine kinase receptor in systemic lupus erythematosus relate to disease activity and nephritis. *Arthritis Research & Therapy*.

[50] Ballantine L., Midgley A., Harris D., Richards E., Burgess S., Beresford M. W. (2015). Increased soluble phagocytic receptors sMer, sTyro3 and sAxl and reduced phagocytosis in juvenile-onset systemic lupus erythematosus. *Pediatric Rheumatology Online Journal*.

[51] Recarte-Pelz P., Tàssies D., Espinosa G. (2013). Vitamin K-dependent proteins GAS6 and protein S and TAM receptors in patients of systemic lupus erythematosus: correlation with common genetic variants and disease activity. *Arthritis Research & Therapy*.

[52] Gong S., Xu Z., Liu Y. (2019). Plasma sMer, sAxl and GAS6 levels correlate with disease activity and severity in lupus nephritis. *European Journal of Clinical Investigation*.

[53] Zhu H., Sun X., Zhu L. (2014). The expression and clinical significance of different forms of Mer receptor tyrosine kinase in systemic lupus erythematosus. *Journal of Immunology Research*.

[54] Ekman C., Jonsen A., Sturfelt G., Bengtsson A. A., Dahlback B. (2011). Plasma concentrations of Gas6 and sAxl correlate with disease activity in systemic lupus erythematosus. *Rheumatology (Oxford)*.

[55] Gheita T. A., Bassyouni I. H., Bassyouni R. H. (2012). Plasma concentrations of growth arrest specific protein 6 and the soluble form of its tyrosine kinase receptor Axl in patients with Systemic lupus erythematosus and Behçets disease. *Journal of Clinical Immunology*.

[56] Suh C. H., Hilliard B., Li S., Merrill J. T., Cohen P. L. (2010). TAM receptor ligands in lupus: protein S but not Gas6 levels reflect disease activity in systemic lupus erythematosus. *Arthritis Research & Therapy*.

[57] Garcia de Frutos P., Fuentes-Prior P., Hurtado B., Sala N. (2007). Molecular basis of protein S deficiency. *Thrombosis and Haemostasis*.

[58] Hafizi S., Dahlback B. (2006). Gas6 and protein S. Vitamin K-dependent ligands for the Axl receptor tyrosine kinase subfamily. *The FEBS Journal*.

[59] Hilliard B. A., Zizzo G., Ulas M., Linan M. K., Schreiter J., Cohen P. L. (2014). Increased expression of Mer tyrosine kinase in circulating dendritic cells and monocytes of lupus patients: correlations with plasma interferon activity and steroid therapy. *Arthritis Research & Therapy*.

[60] Graham D. K., Dawson T. L., Mullaney D. L., Snodgrass H. R., Earp H. S. (1994). Cloning and mRNA expression analysis of a novel human protooncogene, c-mer. *Cell Growth & Differentiation*.

[61] Graham D. K., Bowman G. W., Dawson T. L., Stanford W. L., Earp H. S., Snodgrass H. R. (1995). Cloning and developmental expression analysis of the murine c-mer tyrosine kinase. *Oncogene*.

[62] Ochodnicky P., Lattenist L., Ahdi M. (2017). Increased circulating and urinary levels of soluble TAM receptors in diabetic nephropathy. *The American Journal of Pathology*.

[63] Shao W. H., Zhen Y., Rosenbaum J. (2010). A protective role of Mer receptor tyrosine kinase in nephrotoxic serum-induced nephritis. *Clinical Immunology*.

[64] Fiebeler A., Park J. K., Muller D. N. (2004). Growth arrest specific protein 6/Axl signaling in human inflammatory renal diseases. *American Journal of Kidney Diseases*.

[65] Lee I. J., Hilliard B., Swami A. (2012). Growth arrest-specific gene 6 (Gas6) levels are elevated in patients with chronic renal failure. *Nephrology, Dialysis, Transplantation*.

[66] Yanagita M. (2004). The role of the vitamin K-dependent growth factor Gas6 in glomerular pathophysiology. *Current Opinion in Nephrology and Hypertension*.

[67] Nagai K., Arai H., Yanagita M. (2003). Growth arrest-specific gene 6 is involved in glomerular hypertrophy in the early stage of diabetic nephropathy. *The Journal of Biological Chemistry*.

[68] Nagai K., Miyoshi M., Kake T. (2013). Dual involvement of growth arrest-specific gene 6 in the early phase of human IgA nephropathy. *PLoS One*.

[69] Yanagita M., Arai H., Ishii K. (2001). Gas6 regulates mesangial cell proliferation through Axl in experimental glomerulonephritis. *The American Journal of Pathology*.

[70] Madhusudhan T., Kerlin B. A., Isermann B. (2016). The emerging role of coagulation proteases in kidney disease. *Nature Reviews. Nephrology*.

[71] Li W., Wang J., Ge L., Shan J., Zhang C., Liu J. (2017). Growth arrest-specific protein 6 (Gas6) as a noninvasive biomarker for early detection of diabetic nephropathy. *Clinical and Experimental Hypertension*.

[72] Yanagita M., Ishimoto Y., Arai H. (2002). Essential role of Gas6 for glomerular injury in nephrotoxic nephritis. *The Journal of Clinical Investigation*.

[73] Yanagita M., Ishii K., Ozaki H. (1999). Mechanism of inhibitory effect of warfarin on mesangial cell proliferation. *Journal of the American Society of Nephrology*.

[74] Zhen Y., Priest S. O., Shao W. H. (2016). Opposing roles of tyrosine kinase receptors Mer and Axl determine clinical outcomes in experimental immune-mediated nephritis. *Journal of Immunology*.

[75] Fernandez D., Perl A. (2010). mTOR signaling: a central pathway to pathogenesis in systemic lupus erythematosus?. *Discovery Medicine*.

[76] Wang H., Cnhen S., Chen Y. (2007). The role of Tyro 3 subfamily receptors in the regulation of hemostasis and megakaryocytopoiesis. *Haematologica*.

[77] Choi J. Y., Park H. J., Lee Y. J. (2013). Upregulation of Mer receptor tyrosine kinase signaling attenuated lipopolysaccharide-induced lung inflammation. *The Journal of Pharmacology and Experimental Therapeutics*.

[78] Orme J. J., Du Y., Vanarsa K. (2016). Heightened cleavage of Axl receptor tyrosine kinase by ADAM metalloproteases may contribute to disease pathogenesis in SLE. *Clinical Immunology*.

[79] Criado García O., Sánchez-Corral P., Rodríguez de Córdoba S. (1995). Isoforms of human C4b-binding protein. II. Differential modulation of the C4BPA and C4BPB genes by acute phase cytokines. *Journal of Immunology*.

[80] Wu G., Ma Z., Cheng Y. (2018). Targeting Gas6/TAM in cancer cells and tumor microenvironment. *Molecular Cancer*.

[81] Myers S. H., Brunton V. G., Unciti-Broceta A. (2015). AXL inhibitors in cancer: a medicinal chemistry perspective. *Journal of Medicinal Chemistry*.

[82] Bansal N., Mishra P. J., Stein M., DiPaola R. S., Bertino J. R. (2015). Axl receptor tyrosine kinase is up-regulated in metformin resistant prostate cancer cells. *Oncotarget*.

[83] Fleuren E. D., Hillebrandt-Roeffen M. H., Flucke U. E. (2014). The role of AXL and the in vitro activity of the receptor tyrosine kinase inhibitor BGB324 in Ewing sarcoma. *Oncotarget*.

[84] Giles K. M., Kalinowski F. C., Candy P. A. (2013). Axl mediates acquired resistance of head and neck cancer cells to the epidermal growth factor receptor inhibitor erlotinib. *Molecular Cancer Therapeutics*.

[85] Holland S. J., Pan A., Franci C. (2010). R428, a selective small molecule inhibitor of Axl kinase, blocks tumor spread and prolongs survival in models of metastatic breast cancer. *Cancer Research*.

[86] Du Y., Fu Y., Mohan C. (2008). Experimental anti-GBM nephritis as an analytical tool for studying spontaneous lupus nephritis. *Archivum Immunologiae et Therapiae Experimentalis*.

